# Animal Health and Productivity of Organic Greek Pig Farms: The Current Situation and Prospects for Sustainability

**DOI:** 10.3390/ani13182834

**Published:** 2023-09-06

**Authors:** Georgios I. Papakonstantinou, Ioannis Arsenakis, Aris Pourlis, Vasileios G. Papatsiros

**Affiliations:** 1 Clinic of Medicine, Faculty of Veterinary Medicine, School of Health Sciences, University of Thessaly, Trikalon 224, 43100 Karditsa, Greece; vpapatsiros@vet.uth.gr; 2 Farma Messinias—Provision of Swine Herd Health Services, 12241 Egaleo, Greece; johnarsenakis@hotmail.com; 3 Laboratory of Anatomy Histology and Embryology, Faculty of Veterinary Medicine, School of Health Sciences, University of Thessaly, Trikalon 224, 43100 Karditsa, Greece; apourlis@vet.uth.gr

**Keywords:** organic, farming, Greece, indigenous Greek Black Pig

## Abstract

**Simple Summary:**

This article aims to review organic pig production in Greece. Information on the production, development and health status of organic pig farming in Greece and the potential prospects for sustainability and future development. Among the pig breeds reared on organic farms, the indigenous Greek Black Pig is the most common. Respiratory and parasitic infections are the most common health problems, while high piglet mortality rates are the main welfare issue in Greek organic pig farming. Concerns about how farmers and authorities should utilize the demands of modern consumers are discussed.

**Abstract:**

A review of organic pig production in Greece was carried out. The aim was to present updated information on the production, development and health status of organic pig farming in Greece and potential prospects for sustainability and future development. The indigenous Greek Black Pig is the main breed reared in Greek organic pig farms. All the reasons why Greek Black Pig breeding is ideal for organic farming are mentioned. Furthermore, respiratory and parasitic infections are the most common health problems, while high piglet mortality rates are the main welfare issue in Greek organic pig farming. Concerns about how farmers and authorities should utilize the demands of modern consumers are discussed.

## 1. Introduction

The livestock sector plays a key role in the sustainability of rural economies and ecosystems and is characterized by significant environmental impacts. Due to the increasing global demand for products of animal origin, there is a need to develop modern livestock production systems that are consistent with current trends in food security and sustainability. Organic farming is proposed as an alternative production system to intensive farming and its negative ecological, social and economic consequences [1,2]. Organic agriculture is a production system based on ecological methods, biodiversity and natural sources adapted to local conditions, while intensive agriculture requires the use of methods that may be responsible for various negative impacts [2].

In Mediterranean regions where extensive agriculture is not prevalent, livestock production is the main source of income. The agricultural sector remains crucial to the rural economy, providing many employment opportunities and stabilizing the rural population. Therefore, it is crucial to protect and improve livestock production [3,4]. Different livestock systems have characteristics related to the cultural development of rural areas as they are crucial for the preservation of cultural heritage, including breeds, landscapes and habitats of high ecological value [5]. These characteristics are also associated with the economic development of rural areas. In addition, organic livestock production is characterized by significant environmental benefits, as it is based on environmentally friendly production systems such as extensive, free-range, pasture-based and/or organic livestock production [6].

In the last three decades, organic farming has been increasing annually in European Union (EU) countries, but there are differences between countries. In 2015, the total number of organically raised pigs was 0.978 million animals, with Denmark, France and Germany as the main organic pig producers. However, organic pig farming accounts for less than 1% of the total pig sector in the EU. In 2017, the proportion of organic farming was higher for ruminants (about 5% of the cattle herd and 6% of the sheep and goat herds) than for poultry and pigs (3% and less than 1%, respectively). Organic farming for cattle, sheep and goats is less expensive than poultry and pig farming, which is based on more expensive grain feeding, due to extensive grass feeding [6,7].

In Greece, pig farming is considered one of the most important sectors of industrial livestock production, with a 25% share of domestic meat production and a self-sufficiency rate of about 25–35% (www.minagric.gr/index.php/gr) (accessed on 19 March 2023) [7].

This review aims to present updated information on the production, development and health status of organic pig farming in Greece, as well as on the prospects for sustainability and future development.

## 2. The Organic Pig Population in Greece

Organic production has gradually become a new agricultural sector in Greece. In 2002, national projects for the development of the organic pig sector were launched, mainly in the rural areas of western, central and northern Greece [7]. However, measures are needed to exploit its competitive advantages and expand this sector to a feasible scale [8]. In Greece, the total organic pig population was only 1288 in 2002, while it increased to 175,000 organic pigs in 2007. However, since 2008, the organic pig population has decreased significantly, mainly due to the reduction in funding for national projects triggered by the Greek economic crisis. Combined with the increase in feed costs and insufficient investment in reproduction management, modernization and equipment/barns, the decline in the Greek organic pig population has been significant [7]. From 2013 onwards, the total population seems to be stable, but the total number of breeding animals has decreased (Table 1).

European organic pig farms are based on three main production systems, including raising pigs (a) primarily indoors with access to limited outdoor space (e.g., in Austria and Germany), (b) outdoors year-round with access to temporary housing or permanent buildings (e.g., in Denmark, Italy and the United Kingdom) and (c) a combination of indoor and outdoor space during different production phases or seasons (e.g., in France and Sweden). In organic pig production, piglets may not be weaned before 40 days of age. In Greece, organic pig production includes systems where farrowing takes place mainly indoors, but the sow and her piglets have access to the outdoors at least until weaning (after 40–45 days). Fattening pigs spend most of their lives outdoors year-round but usually have access to permanent basic facilities for feed and water supply.

EU Council regulations (EC-1804/1999 and EC-834/2007) describe the rules for the appropriate risk assessment measures and include the concept of the production of processed feeds and criteria concerning products and substances used (e.g., non-organic feed materials and feed additives) in feed production [7,8,9,10,11]. For example, the main feed requirements for organic pigs are:

(a) Organic swine must be fed organic feed containing primarily organic grains and protein sources preferred by the unit itself or by other units or enterprises subject to the regulations [9,10,11],
(b) feeding is for quality assurance rather than production maximization and must meet the nutritional needs of the animals at various stages of development. Compulsory feeding is prohibited [9,10,11],
(c) Up to 30% of the feed formula of the rations on average may consist of in-conversion feed. If the in-conversion feeds come from a unit of the farm itself, this percentage may be increased to 60% [9,10,11],
(d) GMOs and GMO products shall not be used in the production of feed in organic pig production [9,10,11] and
(e) feed containing minerals, trace elements, vitamins or provitamins shall be of natural origin. If these substances are not available, chemically well-defined analogous substances may be permitted for use in organic swine production. Some premixes and supplementary feeds contain prohibited substances and are therefore not permitted [9,10,11].

The following principles must be observed when using veterinary medicinal products in organic swine production:

(a) phytogenes (e.g., plant extracts, essential oils, etc.), homeopathic products (e.g., plant, animal or mineral substances), trace elements and products are preferable to chemically synthesized allopathic veterinary drugs or antibiotics [9,10,11],
(b) antibiotics, coccidiostats, hormones, drugs, growth promoters and any other substances intended to stimulate growth or production shall not be used in animal nutrition. Hormones, however, may be administered to individual animals as therapeutic veterinary treatment. The use of veterinary vaccines is permitted when a disease has been identified as present in a specific area where the production unit is located [9,10,11].
(c) If the use of phytogenic and homeopathic remedies proves ineffective or is not appropriate to prevent the suffering or distress to the animal, chemical synthetic allopathic veterinary drugs or antibiotics may be used under the responsibility of a veterinarian. When veterinary drugs are used, the type of drug and the details of the corresponding records of the veterinarian (diagnosis, dose and withdrawal period of pharmacological substances, duration of treatment, method of administration) must be clearly indicated [9,10,11].

The Organic Farming Control and Certification System is managed by the Directorate of Organic Farming of the Greek Ministry of Rural Development and Food (HMRD) as the regulatory body, and by the Greek Agricultural Organization “ELGO-DIMITRA” (formerly AGROCERT—Organization for the Certification and Supervision of Agricultural Products), together with approved private inspection bodies. ELGO-DIMITRA is responsible for the implementation of the national policy objectives related to quality assurance and control in livestock and agriculture, based on (a) the assessment, approval and supervision of private certification bodies accredited by the Hellenic Accreditation System (ESYD) and (b) the elaboration and certification of the optional national AGRO standards (AGRO Standards, etc.) (https://www.elgo.gr) (accessed on 22 March 2023).

**Figure 1:**
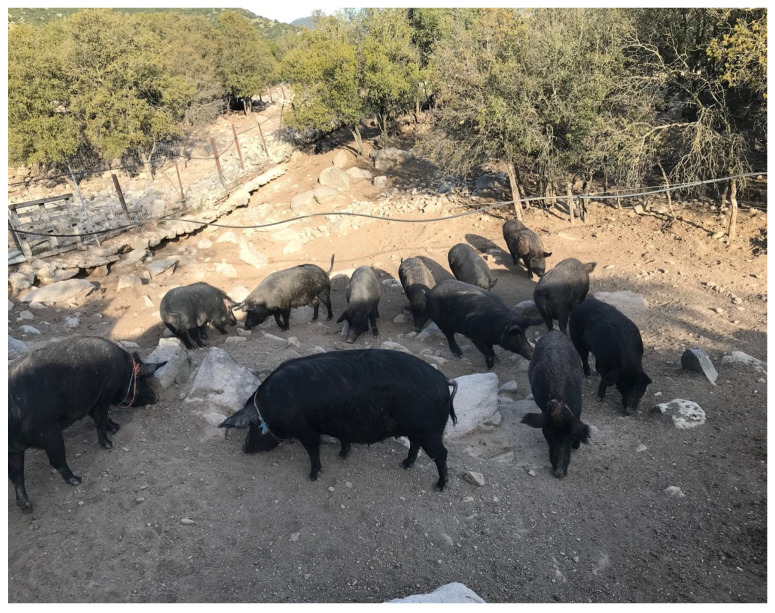
A breeding herd of indigenous Greek Black Pigs. Source: Personal archive of Dr Georgios I. Papakonstantinou.

**Figure 2:**
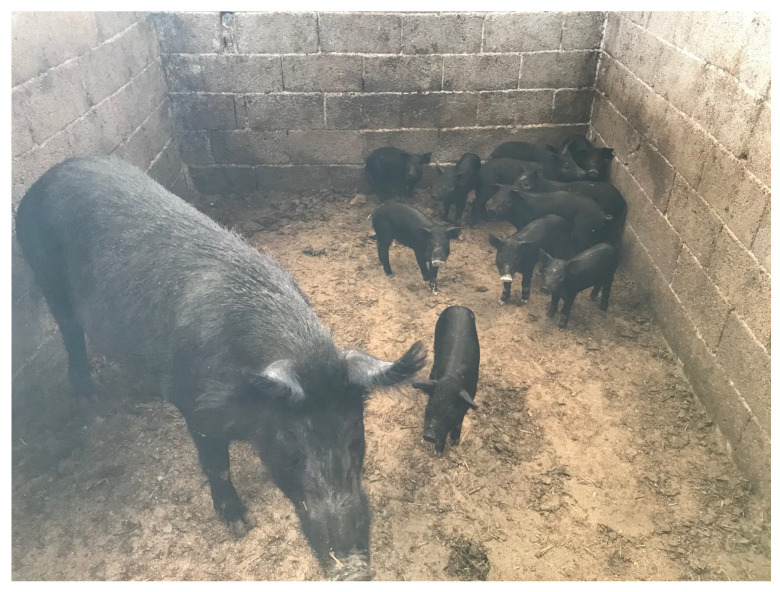
A farrowing sow (indigenous Greek Black Pig) and suckling piglets. Source: Personal archive of Dr Georgios I. Papakonstantinou.

Greek organic pig farms keep either breeding wild pigs (Sus scrofa scrofa); or indigenous pig breeds (Greek Black Pig, Sus scrofa domestica); or hybrids from breeding boars and Greek Black pig with breeding wild pigs; or conventional pig breeds. The indigenous pig breed (Greek Black Pig, also called Greek Pig) is an autochthonous breed raised in Greece, and it is the most common indigenous breed in Greek organic pig farms (Figure 1 and Figure 2). The breed is widely used because of the well-known high quality of its meat and meat products (e.g., sausages and traditionally cured products). Nowadays, the total population is about 3000 animals, mainly kept in central and northern Greece [12]. However, the population of the Greek Black Pig remains small compared to the market demand [13]. Nevertheless, this ancient breed has specific characteristics that can be of great advantage in the implementation of breeding programmes to ensure the long-term survival of this breed [13]. In particular, they exhibit a high degree of genetic variability and adaptability; able to survive in different and harsh environmental conditions of free-range farming [14].

The native Greek Black Pig is an ancient inhabitant of the Mediterranean. In Greece, the breeding of the Greek Black Pig was the main component of extensive free-range farming until 1960. Thus, it was the only domesticated pig [7]. According to Greek mythology, the traces of this breed in the Greek territory date back to 9000 BC. In the years of Homer, it was the breed kept by Evmaios, the mythical swineherd of Odysseus, which fed on the Greek landscapes until a few decades ago. However, the earliest scientific traces of this breed in the Greek territory date back to 5500 BC, when bones of the native Greek Black Pig were found in Sitagrous, Drama [15]. The coat colour of the breed is diverse, as it shows great variations. Black is the predominant colour, but some animals are blackish brown, brown with white grooves, as well as black with white spots. Generally, Greek Black Pigs live in the wild, including oak woodlands. Female Greek Black pigs are characterised by seasonal reproductive activity and two farrowings per year (mean litter size of 8.48 ± 1.94 piglets) [14]. Most organic herds use natural methods of reproduction. However, artificial insemination is allowed, but the administration of hormones to synchronize oestrus or promote growth is prohibited in organic farming. Artificial insemination is the preferred reproductive method in swine production because it reduces the likelihood of breeding sows/chicks with infertile boars. Organic pigs reach sexual maturity relatively late. Therefore, they are weaned at 8 months of age, when their average body weight (BW) is 80–90 kg. The average weight of weaned piglets is estimated to be about 7.69 ± 0.69 kg, and finishing pigs are usually slaughtered at 240–300 days of age, reaching a carcass weight of about 60 kg [14]. In general, the carcass of the Greek Black Pig can provide excellent quality meat and especially cured meat products with excellent organoleptic characteristics. Therefore, Greek consumers distinguish the Greek Black Pig from all other pig breeds raised in the country. Disease resistance and a high degree of adaptation to climatic changes make the Greek Black Pig an ideal breed for rural areas with inhospitable environmental conditions [13].

**Figure 3:**
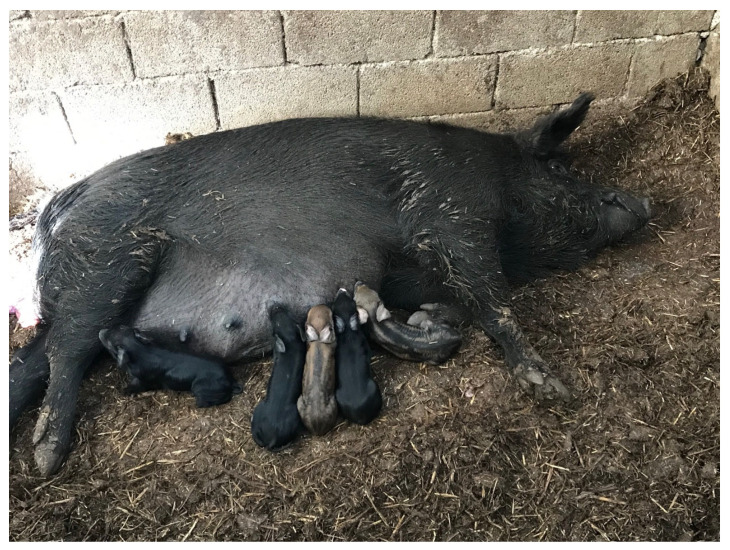
Crossbreed suckling piglets from an indigenous Greek black sow bred with a farmed wild boar. Personal archive of Prof. Vasileios Papatsiros.

One of the main problems of Greek organic pig farming is that breeding animals usually come from non-breeding farms and farmers use unprofessional breeding strategies, including crossbreeding with common breeds from commercial pig farms [13]. Nowadays, the differentiation of Greek breeds is based on phenotypic traits (i.e., coat colour, ear shape, etc.), which poses a great risk not only for the subjective criteria but also for the crossing of breeding animals with common breeds from commercial pig farms (e.g., Duroc) [16]. Crossbreeding between feral pigs and free-ranging pigs or Greek Black Pigs is a common practice in many wild pig farms in Greece [7] (Figure 3). Greek researchers reported a remarkable hybridization with wild boars in the studied wild boar populations of organic farms in northern Greece, probably due to an implemented breeding strategy or uncontrolled reproduction with wild boars [16]. A recent study revealed high genetic variability in the Greek Black Pig breed, suggesting that frequent admixture with both wild pig populations and other conventional pig breeds has occurred [12]. Therefore, any attempt to control and ensure the breeding of a particular native or wild population is done through phenotypic (morphological) characteristics (e.g., coat colour, ear shape, etc.). This fact poses great risks, firstly due to the subjective criteria and secondly because many farmers cross their herds with other improved (domesticated) breeds, which does not make it easy to certify and classify the bred animals as a pure breed or population [16]. For this reason, the establishment of a well-designed plan to maintain the genetic integrity of the Greek Black Pig breed is crucial for its survival and the increase of the overall population.

In Greece, there are no nucleus farms producing replacement gilts for organic farms. Many organic pig farms purchase gilts from conventional farms. According to the current legislation on organic pig farming, all animals must come from organic farms, except in cases necessary for the renewal of the herd, so a 20% share of conventional pigs is allowed (breeding animals from non-breeding farms). A breeding sow or gilts purchased from a conventional farm may give birth to organic piglets if the sow or gilts are kept organically during the last trimester of gestation. However, compliance issues have been raised because many organic pig farmers do not follow the recommended rules [7,16,17]. In addition, gilts purchased from conventional farms are difficult to adapt to outdoor conditions. Producing gilts on free-range farms could provide breeding stock that is optimally adapted to outdoor conditions. In general, local breeds are suitable for organic pig production, especially when crossed with conventional white boar breeds to improve carcass quality (e.g., leanness) [17]. This could be an option for the development of organic pig production in Greece since the total population of local pig breeds remains low.

## 3. Challenges in Organic Pig Production

### 3.1. Animal Health Issues

The most common health problems in the Greek organic pig sector are respiratory infections (e.g., *Mycoplasma hyopneumoniae*) and parasitic infections (mainly due to *Sarcoptes scabei*, Trichuris suis and *Ascaris suum*), high piglet mortality and poor body condition of sows after weaning [7]. In general, the prevalence of helminth infections is higher in free-range pigs than in conventionally housed pigs [18,19]. In addition, organic pig farming is a free-range system that provides important animal welfare benefits but is associated with locomotion problems due to the poor leg health of the commonly used breeds [20].

Most Greek organic pig farms are located in mountainous or semi-mountainous areas where wild boar populations are also present. Farmers report that feral pigs frequently enter their farms, either to access feed or to come into contact with sexually mature females. In our previous study, we reported various seropositivities in farmed feral pigs to seven respiratory and other pathogens such as *Actinobacillus pleuropneumoniae* (*A. pleuropneumoniae*), Aujeszky virus (ADV), Porcine reproductive and respiratory syndrome virus (PRRSV), *Mycoplasma hyopneumoniae* (*M. hyopneumoniae*) and *Erysipelothrix rhusiopathiae* [21]. High seroprevalence was found for *M. hyopneumoniae* and *A. pleuropneumoniae*, and the most common coinfections were *A. pleuropneumoniae* and ADV, and less frequently PRRSV and *M. hyopneumoniae* or PRRSV and *A. pleuropneumoniae*. Based on these results, there is a risk for the above respiratory pathogens in organic swine production, especially when farmers use animals from conventional swine operations as breeding stock.

In general, outdoor pigs have easier and more frequent contact with vectors or reservoirs of wild animals (e.g., birds, rats, foxes or feral pigs). Previous studies investigating the impact of *Trichinella* spp. on free-range pigs in Greece during 2009–2012 have shown that *Trichinella britovi* poses a significant risk [22,23,24]. In particular, 37 out of 12,717 (0.29%) free-ranging pigs which were tested during the period in question, were positive for *Trichinella* spp., came from seven free-range farms in northeastern Greece (Dramas, Evros and Kavala). Moreover, our studies on the prevalence of *Toxoplasma gondii* in pigs revealed that toxoplasmosis could be a potential zoonotic risk factor in Greece [25,26]. Moreover, in organic pig farming there are usually insufficient biosecurity measures to avoid contact with feral pigs. It is known that the increase in feral pig populations is an increased risk factor for the spread of African swine fever (ASF) in Europe [27]. Approximately 47,000 wild boar cases and domestic pig outbreaks of ASF have been reported overall in Europe. The vast majority of these notifications, at over 86%, were made for ASF in wild boar. Less than 14% of the reported events involved ASF in domestic pigs, indicating that wild boars are currently the predominant host for ASF in Europe [27]. The virus can be transmitted either through the direct horizontal route (especially in high densities of wild boar) or through a contaminated environment (ASF-positive carcasses, infected material such as blood and meat from hunting infected animals, or excreta from sick animals). In addition, humans can transmit the virus over long distances through contaminated meat and other by-products such as hides, skulls, tusks, or other hunting trophies [28]. The most recent cases of transmission of the virus from feral pigs to pigs on organic farms have been found in the Czech Republic (Zlin district), Poland (Warsaw) and Hungary (Heves county) [28]. In Greece, the first reported case of ASF was confirmed on February 5, 2020, in a backyard farm (Serres, Northern Greece) [28]. To date, there is no EU legislation defining the categories of free-range pigs and, in general, there is a great lack of information. According to the European Food Safety Authority (EFSA), the baseline risk of free-range pigs for the introduction and spread of ASF is high, but with considerable uncertainty [28]. However, the authorities recommend the implementation of on-farm biosecurity protocols based on the approval of free-range farms [28]. Awareness campaigns play an important role in preventing the spread of disease. These campaigns can include a range of (preventive) information activities conducted in-country, as well as at border crossings for passengers, vehicles, vessels at river ports and airports. To raise awareness, a series of lectures need to be organised for all stakeholders (veterinarians, farmers and hunters), especially in the areas where backyards and free-range farming are predominant, and simple information posters and brochures with the most important basic information need to be distributed to inform and/or remind all free-range pig farmers and their staff. Public media (local radio stations and TV) and local newspapers can also be included in the awareness campaign. Well-known radio programmes and TV, which cover agriculture and livestock, are one of the best and quickest ways to disseminate information about biosecurity recommendations for this type of swine production [29].

The peripartum period (5–7 days before farrowing to 2–3 days after farrowing/early lactation) is a high-risk period for health problems in sows, including mainly reproductive problems such as vulvar discharge and mastitis [7,30,31]. Other reproductive problems include increased heat return rate, poor conception rates, lack of heat synchronization and increased abortion rates [7]. These fertility problems may be related to health or welfare conditions or may be due to poor herd management (e.g., heat detection or insemination practices) or poor professional management [32,33]. Therefore, it is crucial for organic farm owners to take some measures to prevent these health problems in lactating sows. In particular, they should control the barn temperature (the lower and upper critical temperatures for extensive housing on straw and typical feed intake are about 7 °C and 26 °C for lactating sows and 12 °C and 31 °C for dry sows), ensure adequate water supply, examine the sow during and after farrowing and control her rectal temperature. In addition, it is necessary to treat the animals immediately if they show signs of a hard udder and to provide them with separate lying and defecation areas [31,32,33].

Organic farming regulations do not allow the general preventive use of antimicrobial drugs to avoid health problems [34]. Free-range pigs should have a herd health program that includes alternatives to antimicrobials (e.g., acidifiers, prebiotics, probiotics and phytogenics), antiparasitics, vaccines (e.g., against *Escherichia coli*, *M. hyopneumoniae*, PRRSV), and biosecurity and hygiene protocols (e.g., rodent management) to prevent swine and zoonotic diseases, reduce mortality and improve production [34]. Approved parasiticides may be used only when preventive measures have proven ineffective. The use of parasiticides is restricted to breeding animals, and they must be administered before the last trimester of gestation. Treatment of lactating sows or market animals with parasiticides is required when medically necessary, but the sow, her lactating piglets, and the market animals would then lose their organic status [34,35]. In addition, the use of appropriate internal and external biosecurity measures to limit infections circulating in outdoor swine populations is important for public health to minimize the risks of human infection with zoonotic pathogens from the consumption of contaminated organic swine meat [36]. Finally, incorporating herd health programs that prioritize animal welfare and improve the health status of free-range pigs is important [36].

### 3.2. Animal Welfare Issues

One of the main problems of animal welfare in organic pig production is higher piglet mortality compared to conventional pig farming [37,38,39]. The most common causes of piglet mortality are an increased number of stillbirths, the crushing of piglets by the sow, and an increased number of dead piglets with low birth weight due to starvation or hypothermia [30,40]. The number of dead piglets in a litter can be reduced by certain management practices, such as the use of modified farrowing houses for sows, cross-feeding, and better support of sows at birth [40,41]. However, improvements in breeding animal selection could also lead to a decrease in piglet mortality [42]. Schild et al. reported that another factor related to high piglet mortality is the large litter sizes obtained by using highly productive sow lines and suggested a switch to less productive genetics to produce hybrids suitable for organic farming [43]. Chu et al. also suggested that certain sow genotypes and crosses could be useful to reduce piglet mortality in organic herds [44].

Welfare problems could be related to nutrient requirements in organic pig production due to the quantity and quality of feed offered and excessive deterioration in the body condition in lactating sows due to the demands of milk production [30,33]. This is because sows on organic farms are often fed diets of lower nutritional quality, containing low-energy and low-protein components. At the same time, they must endure a longer lactation period compared to conventional swine production and may be exposed to greater thermal challenges due to being kept outdoors. Musculoskeletal problems, such as osteoporosis, could be due to the high demands of milk production during the lactation period. In general, lameness, injuries and sunburn are common health problems on outdoor farms [7,30].

In addition, animal welfare problems could be related to environmental conditions (cold or heat stress) in free-range farms. Depending on geographic location, sows may be exposed to both heat and cold stress throughout the year. Lactating sows are most affected by heat stress (higher feed intake requirements and metabolic activity for milk production), while dry sows are usually affected by cold stress because they receive a restricted diet. According to recent studies, climate change in Greece is expected to lead to warmer temperatures characterized by an increased number of hot days with temperatures of >35 °C during daytime hours and up to 20 °C during nighttime hours for more than 50 days per year in most areas [45].

## 4. Future Directions and Prospects for Sustainability

**Figure 4:**
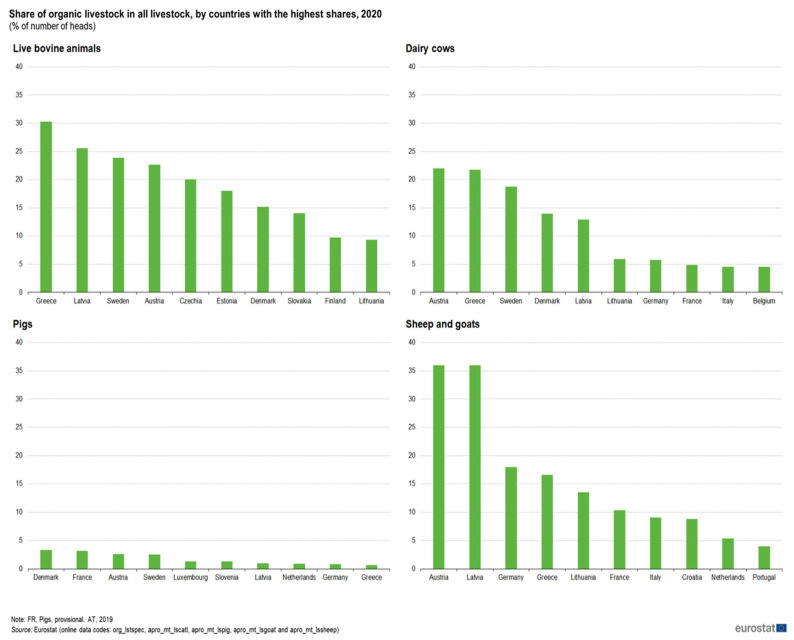
Share of organic livestock in all livestock sectors by countries with the highest shares, 2020 (% of the number of heads) (https://ec.europa.eu/eurostat/statistics-explained/index.php?title=File:Fig6_Share_of_organic_livestock_in_all_livestock,_by_countries_with_the_highest_shares,_2020_(%25_of_number_of_heads).png#filelinks) (accessed on 28 March 2023).

The efficient and inexpensive supply of products of animal origin is no longer sufficient for the social acceptance of industrial animal husbandry [46,47,48,49]. Today, social acceptance of intensive animal husbandry is based not only on economic benefits but also on ethical requirements [50,51,52]. Organic pig farming meets economic and environmental criteria and as such it could be a profitable industry and an environmentally friendly production system. The proportion of organic pig production in the total livestock production of the EU is one of the lowest, while organic production of cattle and small ruminants is one of the largest (Figure 4).

Pork self-sufficiency varies among European countries, ranging from 30% in Greece to >400% in Denmark (IFIP, 2021). Pork consumption in Europe is mainly (up to 75% in some EU countries) based on a variety of processed hot or cold meat products (e.g., dry-cured, cooked, dried hams, sausages, etc.). However, this wide variety of meat products depends on the different and specific requirements of the food supply chain actors [52]. Greece imports mainly unprocessed and fresh carcass cuts from EU Member States to be processed into certain traditional products with a large market share (e.g., Greek gyros).

The demand for organic products in the European market is increasing due to the growing interest in valuable nutrients and high-quality agricultural products, as well as the increasing sensitivity to environmental issues. Research in the EU has shown that consumers in Greece and other Mediterranean countries (Italy, Spain and Portugal) pay more attention to the environmental impact of their food than in other European countries (such as Belgium, Germany, Lithuania, the Netherlands and Slovakia). However, a recent study investigating consumer perceptions and preferences in Greece found that awareness of the daily consumption of organic food is only average and the price is too high [53]. Although Greek consumers indicated that organic food was primarily safer, they were not strongly in favour of its environmental aspects. According to the results of this review, information campaigns about the benefits of organic food are crucial to raising consumer awareness about environmental issues [54]. Regarding Greek consumers’ preferences for pork and pork products, they seem to be very interested in information about these products [55]. This consumer preference also correlates with a high interest in a traceability system that would benefit the development of organic pig farming [55]. Organic pig production should implement traceability systems from “farm to fork” to meet modern consumer demands and increase the attractiveness of its environmentally friendly products.

Especially in Greece, there is a lack of agricultural guarantee systems that complement each other in terms of presentation on meat packaging for consumers, both in terms of the type of labelling and the husbandry system [56]. One example is the UK pig industry, where labels such as “organic” and “RSPCA-assured” are combined with the type of housing system as indicated on meat packages (i.e., indoor, outdoor, combined indoor/outdoor, slatted floors, straw). Akaichi et al. reported that consumer demand for organic pork increases when consumers are offered pork products labelled with agricultural guarantee systems [41].

In Europe, chicken and pork are mainly produced industrially, while ruminants are mainly raised on grassland or in mixed systems. In Europe, agroforestry has a long history in traditional free-range pig farming. This practice is based on feeding pigs with forage from natural sources in oak (*Quercus* sp.) and beech (*Fagus sylvatica*) forests and with fallen fruit from orchards in autumn [57]. Greece has a great advantage in this agroforestry practice, having the second-largest extension of agroforestry with livestock after Spain (5.5 million ha) [58]. Organic pig farming could be a positive factor to take advantage of the expansion of agroforestry land or forests and boost the local economy. 

The “Farm to Fork Strategy” and the EU’s published “2021–2027 Organic Action Plan” demonstrate that the organic sector could play a key role in achieving the goals of the European Green Deal for the food industry, and therefore that organic farming must be further supported by the EU. However, crucial for the future development of the organic pig sector is the ability to maintain “a spirit of cooperation among all interested groups and a high level of trust”, as the relationship between farmers, consumers and processors is influenced by several socio-economic and “psychological” parameters [7]. Pfeifer et al. studied the resilience of 18 pig organic pig farmers across Europe to market disruptions and reported that one of their challenges was direct marketing, which resulted in higher labour costs [59]. Low self-sufficiency in forage and lack of available land were other factors that affected resilience. Low yield per hectare, including the yield of certified crops used to feed the pigs, was also cited as one of the reasons preventing improvement in the greenhouse gas impact of organic pork production systems [57]. In Greece, a new government initiative addressed the definition of grazing areas for livestock used in food production, including pigs. This will complement future national strategies to more accurately determine the contribution of organic pig production to greenhouse gas emissions in Greece.

In addition, the implementation of a health management and disease prevention program based on a hazard analysis and critical control points (HACCP) approach can provide the basis for ensuring food safety and the high quality of organic pork through regular monitoring of disease risk factors. The development of a HACCP system on organic farms requires the quantification of risk factors through epidemiological studies or, alternatively, by a panel of experts. HACCP systems are characterized by continuous monitoring of risk factors as part of farm health management [25,35]. The overall health status of the herd could also be improved through the implementation of good agricultural practices, such as hygiene measures, quarantine facilities for newly purchased animals and good rodent control measures. Disease surveillance through records at the slaughterhouse or through blood and faecal sampling, followed by standard measures in case of problems, should be included in the farm health management program [22,24,34]. The application of integrated veterinary management on organic swine farms will ensure the quality and safety of the organic pork produced and contribute to the competitiveness of this particular meat market. Financial and scientific support, as well as the implementation of training programs (especially for young farmers) for Greek farmers could improve the current conditions in organic pig farming [22,24,34].

Therefore, it is of great importance that organic pig farming in Greece is better organized, advised and supported by livestock experts. Financial and scientific support, as well as the implementation of training programs for Greek farmers (especially for young farmers), could improve the current conditions in organic pig farming. In addition, awareness-raising campaigns such as lectures or/and distribution of brochures with basic information should be organized.

## 5. Conclusions

Organic pig farming has remained stable during the last few years but has optimistic potential in the European market given increased demands for the more environmentally friendly production of pork. Greece also has advantages for organic pig production, based on its climate and environmental characteristics that could boost lower production costs. However, owners of free-range pig farms suffer from various productivity losses (organic pig health and welfare issues), resulting in financial losses and low capacity to cover consumer demands. Therefore, it is of great importance that organic pig farming in Greece is better organized, advised and supported by veterinarians and animal scientists.

## Figures and Tables

**Table 1:** Organic pig population in Greece from 2012 to 2020.

Year	Organic Pig Population			
	Total Number of Pigs	Fatteners	Breeding Stock	Other Ages (Suckling/Weaning Piglets)
2012	6292	1892	1933	2467
2013	4797	1770	1406	1621
2014	4664	1512	1268	1884
2015	4203	1469	1004	1730
2016	4710	1977	1005	1728
2017	4434	1928	975	1531
2018	4746	2275	958	1513
2019	4994	2212	1008	1774
2020	5075	2379	1004	1692

Source: Hellenic Ministry of Rural Development and Food.

## Data Availability

Not applicable.
